# Professionalism and Challenges of Indonesian Caregivers for Older Adults in Taiwan: A Qualitative Study

**DOI:** 10.1093/geroni/igaf122.3322

**Published:** 2025-12-31

**Authors:** Fang-Lin Kuo, Herry Susanto, Widiyaningsih Widiyaningsih

**Affiliations:** National Health Research Institutes, Zhunan Town, Miaoli, Taiwan (Republic of China); Universitas Islam Sultan Agung, Semarang, Jawa Tengah, Indonesia; Taipei Medical University, Taipei City, Taipei, Taiwan (Republic of China)

## Abstract

Indonesian caregivers are the largest group of migrant workers in Taiwan and play a critical role in the country’s social welfare sector, particularly in caring for older adults. Despite their significant contributions, their professionalism and lived caregiving experiences remain underexplored. This study investigated the motivations, challenges, and essential skills required for effective caregiving among Indonesian migrant workers. A qualitative phenomenological study was conducted using semi-structured interviews with 10 female caregivers who had worked in Taiwan for an average of 6.9 years. Interviews explored participants’ perspectives on caregiving responsibilities, communication, conflict resolution, emotional resilience, and professional development. Thematic analysis identified three key themes. First, financial and family-related factors serve as primary motivations for pursuing caregiving work in Taiwan, shaping caregivers’ long-term career decisions. Second, caregivers identified essential qualities for professional caregiving, including physical endurance, adaptability, mental resilience, and lifelong learning. Third, participants reported significant challenges in their caregiving roles, including language barriers, conflicts with older adults’ families and job expectations exceeding initial agreements. Despite these difficulties, caregivers adopted coping strategies such as engaging in social activities, using technology for self-protection and communication, and relying on religious practices for emotional support. Findings demonstrate the complexity of caregiving among migrant workers and the need for enhanced training in language skills, emergency response, and specialized geriatric care techniques. Addressing these areas can improve caregivers’ well-being and the quality of care provided to older adults in Taiwan. These insights contribute to policy recommendations for better support systems and workforce development initiatives tailored to migrant caregivers.

